# Two New Phenolic Glucosides from *Lagerstroemia speciosa*

**DOI:** 10.3390/molecules20034483

**Published:** 2015-03-10

**Authors:** Janggyoo Choi, Jae Youl Cho, Soo Jung Choi, Heejin Jeon, Young Dong Kim, Khin Myo Htwe, Young Won Chin, Woo Shin Lee, Jinwoong Kim, Kee Dong Yoon

**Affiliations:** 1 College of Pharmacy and Research Institute of Pharmaceutical Science, Seoul National University, Seoul 151-742, Korea; E-Mails: dashutiao@naver.com (J.C.); jwkim@snu.ac.kr (J.K.); 2 Department of Genetic Engineering, Sungkyunkwan University, Suwon 440-746, Korea; E-Mail: jaecho@skku.edu; 3 College of Pharmacy, The Catholic University of Korea, Bucheon 420-743, Korea; E-Mails: wjd6694@naver.com (S.J.C.); tkghs35@naver.com (H.J.); 4 Department of Life Science, Hallym University, Chuncheon 200-702, Korea; E-Mail: ydkim@hallym.ac.kr; 5 Popa mountain park, Forest Department, Kyaukpadaung Township, Mandalay Division, Myanmar; E-Mail: khinmyohtwe007@gmail.com; 6 College of Pharmacy and RFIND-BKplus Team, Dongguk University-Seoul, 32 Dongguk-lo, Ilsan dong-gu, Goyang 410-820, Korea; E-Mail: f2744@dongguk.edu; 7 Department of Forest Sciences, Seoul National University, Seoul 151-921, Korea; E-Mail: krane@snu.ac.kr

**Keywords:** *Lagerstroemia speciosa*, phenolic glucoside, inhibitory activities against nitric oxide production

## Abstract

Two new phenolic glucosides, 1-*O*-benzyl-6-*O*-*E*-caffeoyl-β-d-glucopyranoside and 1-*O*-(7*S*,8*R*)-guaiacylglycerol-(6-*O*-*E*-caffeoyl)-β-d-glucopyranoside, were isolated from the aerial parts of *Lagerstroemia speciosa*, along with ten known compounds. The structures of the isolated compounds were determined based on 1D- and 2D-NMR, Q-TOF MS and optical rotation spectroscopic data. All of the compounds showed moderate inhibitory activities against nitric oxide production in lipopolysaccharide-treated RAW264.7 cells, with IC_50_ values of 69.5–83.3 μM.

## 1. Introduction

*Lagerstroemia speciosa* L. Pers., which belongs to the Lythraceae family, is a tropical plant distributed in areas of Southeast Asia, including the Philippines, Vietnam, Myanmar and southern China. In these regions, *L. speciosa* is commonly called banaba, and it has been traditionally used for the treatment of diabetes, obesity and kidney malfunction [1,2].

Earlier phytochemical investigations have revealed that *L. speciosa* possessed triterpenes and sterols [3,4], phenolic compounds, including ellagic and gallic acid [5], ellagitannins and several flavonoids [6,7]. *In vivo* and *in vitro* studies indicated that banaba extract shows anti-α-glucosidase activity [3], anti-HIV-1 protease and reverse transcriptase activities [5], anti-human rhinovirus activity [6], hypoglycemic activity [7,8] and free radical scavenging and anti-inflammatory effects [9]. For further phytochemical investigation of *L. speciosa*, diverse column chromatography methods were performed to isolate two novel phenolic compounds, 1-*O*-benzyl-6-*O*-*E*-caffeoyl-β-D-glucopyranoside **1** and 1-*O*-(7*S*,8*R*)-guaiacylglycerol-(6-*O*-*E*-caffeoyl)-β-D-glucopyranoside **2**, along with ten known compounds, **3**–**12**. In addition, the inhibitory activities of these isolated compounds against nitric oxide (NO) production in lipopolysaccharide (LPS)-treated RAW 264.7 cells were evaluated.

## 2. Results and Discussion

### 2.1. Structural Elucidation of Isolated Compounds

**Figure 1:**
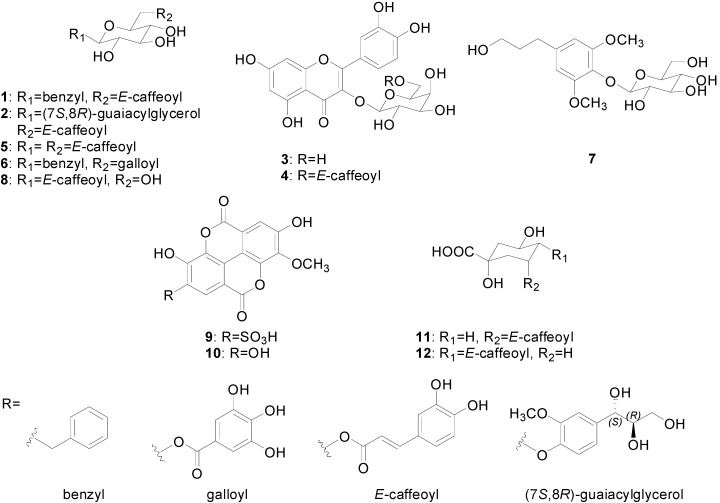
Chemical structures of Compounds **1**–**12** from *L. speciosa.*

The structure elucidation of two new compounds (**1** and **2**) was accomplished by interpretation of the spectroscopic data, including 1D- and 2D-NMR, Q-TOF MS and optical rotation data. The ten known compounds were determined to be quercetin-3-*O*-β-d-galactopyranoside **3** [10], quercetin-3-*O*-(6''-*O*-*E*-caffeoyl)-β-d-galactopyranoside **4** [11], 1,6-di-*O*-*E*-caffeoyl-β-d-glucopyranoside **5** [12], benzyl-6'-*O*-galloyl-β-d-glucopyranoside **6** [13], dihydrosyringin **7** [14], 1-*O*-*E-*caffeoyl-β-d-glucopyranoside **8** [15], 3-*O*-methylellagic acid 4'-sulfate **9** [7], 3-*O*-methylellagic acid **10** [7], chlorogenic acid **11** [16] and cryptochlorogenic acid **12** [16] (Figure 1). To the best of our knowledge, the known eight compounds (**3**–**8** and **11**–**12**) were isolated for the first time from *L. speciosa*.

**Figure 2:**
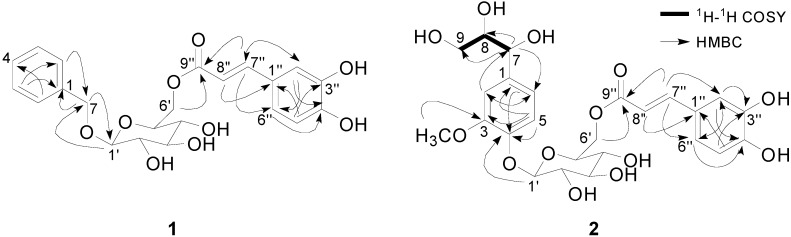
Key ^1^H-^1^H COSY and HMBC correlations for Compounds **1** and **2**.

Compound **1** was isolated as an amorphous brownish powder, and its molecular formula was assigned as C_22_H_24_O_9_ from the [M+H]^+^ and [M+Na]^+^ ion peaks at *m/z* 433.1506 (calcd. for C_22_H_25_O_9_: 433.1499) and 455.1328 (calcd. for C_22_H_24_O_9_Na: 455.1318), respectively, by Q-TOF MS. The ^1^H-NMR spectrum showed resonances corresponding to a benzyl moiety (δ_H_ 7.38 (2H, d, *J* = 7.3 Hz; H-2, H-6), 7.31 (2H, t, *J* = 7.3 Hz; H-2, H-6), 7.27 (1H, t, *J* = 7.3 Hz; H-4), 4.76 (1H, d, *J* = 12.1 Hz; H-7a), 4.58 (1H, d, *J* = 12.1 Hz; H-7b)) and an *E*-caffeoyl residue (δ_H_ 7.51 (1H, d, *J* = 15.9 Hz; H-7''), 7.04 (1H, br s; H-2''), 6.99 (1H, br d, *J* = 7.2 Hz; H-6''), 6.72 (1H, d, *J* = 7.8 Hz; H-5''), and 6.30 (1H, d, *J* = 15.9 Hz; H-8'')), as well as a signal assigned to the anomeric proton of the glucosyl moiety (δ_H_ 4.28 (1H, d, *J* = 7.9 Hz; H-1')) (Table 1). The ^13^C-NMR spectrum of **1** showed six glucopyranosyl signals at δ_C_ 102.0, 76.5, 73.8, 73.4, 70.1 and 63.5, and the configuration was confirmed as the β-form from the coupling constant. HMBC correlations of H-7 (δ_H_ 4.76 and 4.58) to C-1' (δ_C_ 102.0) and H-6' (δ_H_ 4.41 and 4.19) to C-9'' (δ_C_ 166.6) indicated that **1** is a caffeic acid ester of 1-*O*-benzyl-β-d-glucopyranoside (Figure 2). Based on the spectroscopic evidence above, **1** was determined to be 1-*O*-benzyl-6-*O*-*E*-caffeoyl-β-d-glucopyranoside.

The positive Q-TOF MS spectrum of Compound **2** showed a sodiated pseudomolecular ion peak at *m*/*z* 561.1595 (calcd. for C_25_H_30_O_13_Na: 561.1584); thus, its molecular formula was determined to be C_25_H_30_O_13_. The ^1^H- and ^13^C-NMR spectra of **2** indicated the presence of 6-*O*-*E*-caffeoyl-β-d-glucopyranoside, similar to the case of Compound **1**. The difference between **1** and **2** is that the guaiacylglycerol moiety is linked to a glucosyl group instead of a benzyl moiety. 1D- and 2D-NMR spectra, including ^1^H-^1^H COSY and HMBC spectra, revealed the presence of a guaiacylglycerol skeleton. The ^1^H-NMR spectrum indicated the presence of a 1,3,4-trisubstituted aromatic ring (δ_H_ 7.09 (1H, d, *J* = 8.3 Hz; H-5), 7.08 (1H, d, *J* = 1.8 Hz; H-2) and 6.83 (1H, dd, *J* = 8.3, 1.8 Hz; H-6)), a methene group (δ_H_ 3.48 (1H, m; H-9a) and 3.35 (1H, m; H-9b)), two methine protons (δ_H_ 4.52 (1H, d, *J* = 4.2 Hz; H-7) and 3.62 (1H, m; H-8)) and a methoxy group (δ_H_ 3.87 (3H, s)) (Table 1). A ^1^H-^1^H COSY experiment revealed the partial structure of -CH(7)-CH(8)-CH_2_(9), and the HMBC correlation of H-1' (δ_H_ 4.87) to C-4 (δ_C_ 147.0) revealed the C4-*O*-C1' connectivity between the 6-*O*-*E*-caffeoyl-β-d-glucopyranoside and guaiacylglycerol moieties (Figure 2). The small *J*_H7,H8_ coupling constant (*J* = 4.2 Hz) in the ^1^H-NMR spectrum of **2** suggested that C-7 and C-8 were in the *erythro* configuration (*J*_H7,H8_ ≤ 4.4 Hz); a relatively large coupling constant (*J*_H7,H8_ ≥ 6.0 Hz) would be expected for the *threo* configuration [17]. Enzymatic hydrolysis of **2** afforded **2a**, which had a pseudomolecular ion peak at *m*/*z* 237.0743, [M+Na]^+^ (calcd. for C_10_H_14_O_5_Na: 237.0739), in the Q-TOF MS data, and an optical rotation of ${\left[\alpha \right]}_{D}^{22}\;=\;+11.2,$ which demonstrated the presence of the (7*S*,8*R*)-guaiacylglycerol unit [17,18]. According to the spectroscopic data above, Compound **2** was assigned as 1-*O*-(7*S*,8*R*)-guaiacylglycerol-(6-*O*-*E*-caffeoyl)-β-d-glucopyranoside.

**Table 1:** ^1^H- (500 MHz, δ ppm, *J* in Hz) and ^13^C-NMR (125 MHz, δ ppm) of Compounds **1** and **2** in CD_3_OD.

1			2		
Position	δ_H_ (*J* in Hz)	δ_C_	Position	δ_H_ (*J* in Hz)	δ_C_
1		137.8	1		138.7
2,6	7.38, d (7.3)	127.8	2	7.08, d, (1.8)	112.5
3,5	7.31, t (7.3)	128.1	3		150.8
4	7.27, t (7.3)	127.4	4		147.0
7a	4.76, d (12.1)	69.6	5	7.09, d (8.3)	117.8
7b	4.58, d (12.1)		6	6.83, dd (8.3, 1.8)	120.7
1'	4.28, d (7.9)	102.0	7	4.52, d (4.2)	75.2
2'	3.07, m	73.4	8	3.62, m	77.5
3'	3.17, m	76.5	9a	3.48, m	64.4
4'	3.16, m	70.1	9b	3.35, m	
5'	3.40, m	73.8	1'	4.87, d (7.6)	102.9
6'a	4.41, brd (11.7)	63.5	2'	3.52, m	75.0
6'b	4.19, dd (11.7, 6.6)		3'	3.49, m	77.9
1''		125.1	4'	3.41, m	72.0
2''	7.04, brs	114.5	5'	3.68, m	75.8
3''		145.5	6'a	4.53, dd (11.9, 2.1)	64.8
4''		149.2	6'b	4.32, dd (11.9, 6.9)	
5''	6.72, d (7.8)	115.7	1''		127.9
6''	6.99, d (7.2)	121.5	2''	7.06, d (2.0)	115.4
7''	7.51, d (15.9)	145.4	3''		147.3
8''	6.30, d (15.9)	113.4	4''		149.9
9''		166.6	5''	6.80, d (8.2)	116.8
			6''	6.96, dd (8.2, 2.0)	123.2
			7''	7.57, d (15.9)	147.3
			8''	6.28, d (15.9)	115.1
			9''		169.1
			OCH_3_	3.87, s	56.8

### 2.2. Inhibitory Activity against NO Production

The inhibitory effects of **1**–**12** against LPS-induced NO production in RAW264.7 cells were evaluated: all isolates showed moderate inhibitory activities, with IC_50_ values of 69.5–83.3 μM (Table 2).

**Table 2:** Inhibitory effects of Compounds **1**–**12** against lipopolysaccharide-induced nitric oxide production in RAW 264.7 cells.

Compound	Cell Viability IC_50_ (μM)	NO Inhibition IC_50_ (μM)
**1**	>100	81.8 ± 1.3
**2**	87.7 ± 3.5	71.1 ± 1.9
**3**	>100	81.9 ± 1.9
**4**	>100	83.3 ± 0.9
**5**	>100	69.5 ± 1.4
**6**	99.0 ± 0.4	73.8 ± 0.5
**7**	>100	76.2 ± 0.6
**8**	84.8 ± 5.0	70.7 ± 1.2
**9**	>100	79.1 ± 1.3
**10**	>100	78.0 ± 2.3
**11**	>100	81.2 ± 0.7
**12**	>100	81.1 ± 0.5

## 3. Experimental Section

### 3.1. General Experimental Procedures

^1^H- and ^13^C-NMR spectra were recorded on an AscendTM 500 spectrometer (Bruker, Germany). Mass spectra were obtained with a 6530 ESI-Q-TOF MS instrument (Agilent Technologies, Santa Clara, CA, USA). UV spectra were recorded with a UV-1800 spectrometer (Shimadzu, Japan). Optical rotations were measured with a P-2000 polarimeter (Jasco, Tokyo, Japan). A Gilson preparative HPLC system (Gilson, Middelton, WI, USA), equipped with binary pumps, a UV/Vis-155 detector and a GX-271 liquid handler, was used to isolate the compounds. Semi-preparative high-performance countercurrent chromatography (HPCCC) was performed with a Dynamic Extractions Spectrum HPCCC instrument that contained two experimental bobbins. One bobbin possessed a semi-preparative coil of 70.5 mL, 3.2 mm inner diameter. The other bobbin contained a semi-preparative coil of 70.5 mL. The β-values ranged from 0.64 to 0.81 for the analytical column and from 0.52 to 0.86 for the semi-preparative column. Organic solvents used for chromatography were of analytical grade and obtained from Daejung Chemical and Metals (Gyunggido, Korea). HPLC-grade solvents, including methanol and water, were purchased from Fisher Scientific Korea (Seoul, Korea). Silica gel 60 and RP-C_18_ silica gel were purchased from Merck (Kenilworth, NJ, USA), and Sephadex LH-20 was obtained from Pharmacia Co. (Stockholm, Sweden). Analytical HPLC was performed with a YMC-Pack ODS-A column (250 × 20 mm ID, 5 μm; YMC, Japan). *N*_ω_-nitro-l-arginine methyl ester hydrochloride (l-NAME) and LPS (*Escherichia coli* 0111:B4) were purchased from Sigma Chemical Co. (St. Louis, MO, USA).

### 3.2. Plant Material

Leaves and twigs of *L. speciosa* were collected at Popa Mountain National Park (Mandalay, Myanmar) in August 2011, and identified by Young Dong Kim (Hallym University, Chuncheon, Korea). A voucher specimen (No. MM-0097) was deposited at the herbarium of the National Institute of Biological Research (Incheon, Korea).

### 3.3. Extraction and Isolation

The dried and ground aerial parts of *L. speciosa* (534 g) were extracted with methanol in an ultrasonic bath (3 h × 3 times) and evaporated under reduced pressure to give a methanol extract (47 g). The methanol extract was suspended in water and successively partitioned to give *n*-hexane (11 g), ethyl acetate (8 g) and *n*-butanol (10 g) soluble extracts. The ethyl acetate fraction was subjected to Diaion HP-20 column chromatography (CC) with gradient elution (20%–100% methanol) to give eight subfractions (Fraction E1-1–Fraction E1-8). Fraction E1-4 (760 mg) was subjected to HPCCC (*n*-hexane/ethyl acetate/methanol/water, 2:8:2:8 v/v; 1,600 rpm; 3 mL/min) to yield four subfractions (Fraction E1-4-1–Fraction E1-4-4). Compound **3** (14 mg) was obtained from E1-4-3 by HPLC on ODS silica gel (ODS HPLC) (eluent, 47:53 *v*/*v* methanol/water mixture). Compounds **4** (2.8 mg) and **8** (1.4 mg) were isolated from Fraction E1-7 by Sephadex LH-20 CC (eluent: methanol), followed by repetitive reversed-phase HPLC (RP-HPLC) with gradient elution (methanol-water, 15:85 → 60:40, v/v). Fraction E1-6 was subjected to Sephadex LH-20 CC (eluent: methanol), followed by repetitive ODS HPLC with gradient elution using a methanol-water mixture (20%–65% methanol) to obtain Compounds **1** (1.7 mg), **2** (2.8 mg), **5** (2.1 mg) and **6** (4.2 mg).

The *n*-butanol fraction was fractionated into eleven subfractions (Fraction B1–Fraction B11) by gravity-driven column chromatography with gradient elution (chloroform–methanol mixture, 9:1 → 10:0, v/v). Fraction B3 was subjected to Sephadex LH-20 CC, followed by RP-HPLC with gradient elution (methanol-water mixture, 15%–50% methanol) to give Compound **7** (1.1 mg). Fraction B5 was subjected to HPCCC (ethyl acetate/*n*-butanol/water system, 6:4:10 v/v; 1,600 rpm; 3 mL/min); the lower phase was used as the mobile phase to obtain six subfractions (Fraction B5-1–B5-6). Compound **8** (5.8 mg) was isolated from Fraction B5-3 by RP-HPLC with gradient elution (methanol-water mixture, 15%–60% methanol). Fraction B7 was subjected to normal-phase column chromatography to afford five subfractions (Fraction B7-1–Fraction B7-5); then, Fraction B7-3 was subjected to RP-HPLC to give Compounds **9** (6.0 mg) and **10** (4.1 mg). Compounds **11** (5.7 mg) and **12** (1.7 mg) were obtained from Fraction B7-5 after RP-HPLC with gradient elution (methanol-water mixture, 10:90 → 50:50, v/v). The structures of the isolated compounds were determined by 1D- and 2D-NMR spectroscopy, MS analysis and comparison with the corresponding spectroscopic data in the literature.

### 3.4. Enzymatic Hydrolysis of Compound **2**

Compound **2** (1.5 mg) was dissolved in H_2_O (2 mL) and hydrolyzed with β-glucosidase (4 mg, from almonds, Sigma-Aldrich) at 37 °C for 72 h. The reaction mixture was evaporated under reduced pressure, and the residue was dissolved in methanol (2 mL). The methanol solution was centrifuged at 10,000 rpm, and then, the supernatant was subjected to RP-HPLC (10:90 MeCN/H_2_O) to yield **2a** (0.24 mg).

### 3.5. Measurement of NO Production 

RAW264.7 cells were plated at 1 × 10^6^ cells/mL and incubated for 18 h. After incubation, the cells were treated with Compounds **1**–**12** for 30 min and then continuously incubated with LPS (1.0 μg/mL) for 24 h. The inhibitory effects of the isolated compounds on NO production were evaluated by using the Griess reagent and an EIA kit to determine the NO levels, as described previously [19].

### 3.6. Cell Viability Test

RAW264.7 cells were plated at 1 × 10^6^ cells/mL and incubated for 18 h followed by the addition of Compounds **1**–**12** to the cells (25 to 150 μM), and the cells were incubated for 24 h. The cytotoxicity was evaluated by an MTT (3-(4,5-dimethylthiazol-2-yl)-2,5-diphenyltetrazolium bromide) assay. The 10 μM of MTT solution (10 mg/mL in phosphate buffered saline, pH 7.4) were added to the cells 3 h prior to the end of the culture period. The incubation was terminated by the addition of 15% sodium dodecyl sulfate to each well to solubilize the formazan. The absorbance was measured by a microplate reader at 570 nm (OD_570-630_).

## Supplementary Materials

Supplementary materials can be accessed at: http://www.mdpi.com/1420-3049/20/03/4483/s1.
