# Supplementary material for: Two New Phenolic Glucosides from Lagerstroemia speciosa
Source: Molecules. 2015 Mar 10;20(3):4483–91. doi: 10.3390/molecules20034483 (PMC6272251; doi:10.3390/molecules20034483)
Supplement: Supplementary file 1 [file molecules-20-04483-s001.pdf]

Supplementary Materials

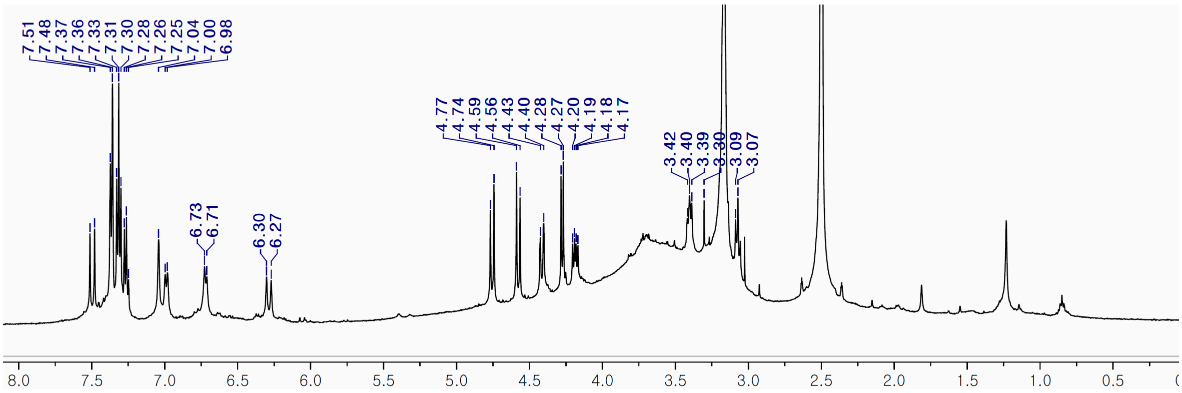

Figure S1. ^{1}H-NMR spectrum of compound **1** (CD_3OD, 500 MHz, δ ppm).

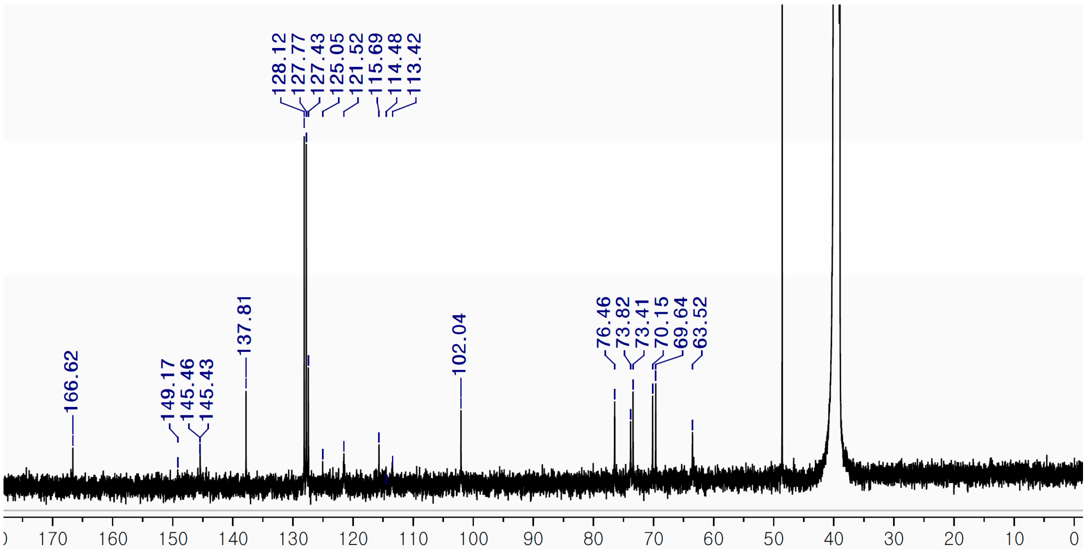

Figure S2. ^{13}C-NMR spectrum of compound **1** (CD_3OD, 125 MHz, δ ppm).

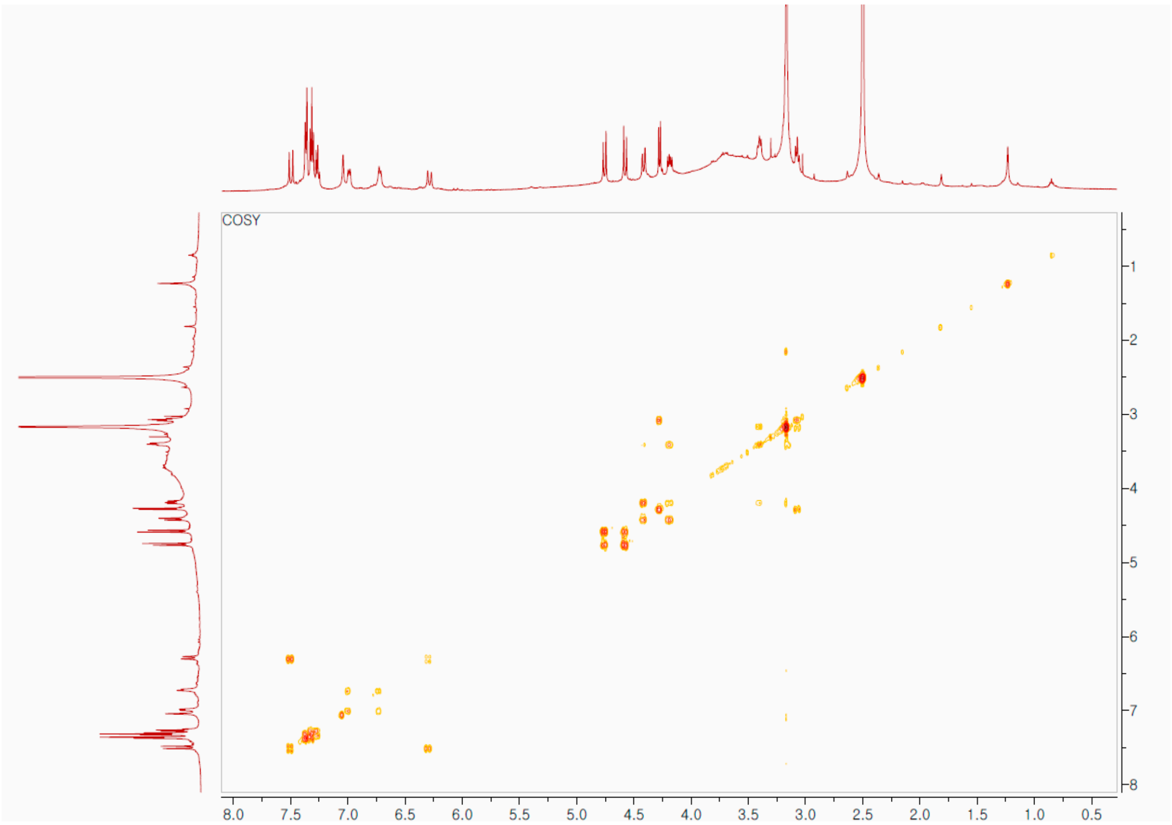

Figure S3. ^{1}H-^{1}H COSY spectrum of compound **1** (CD_3OD, δ ppm).

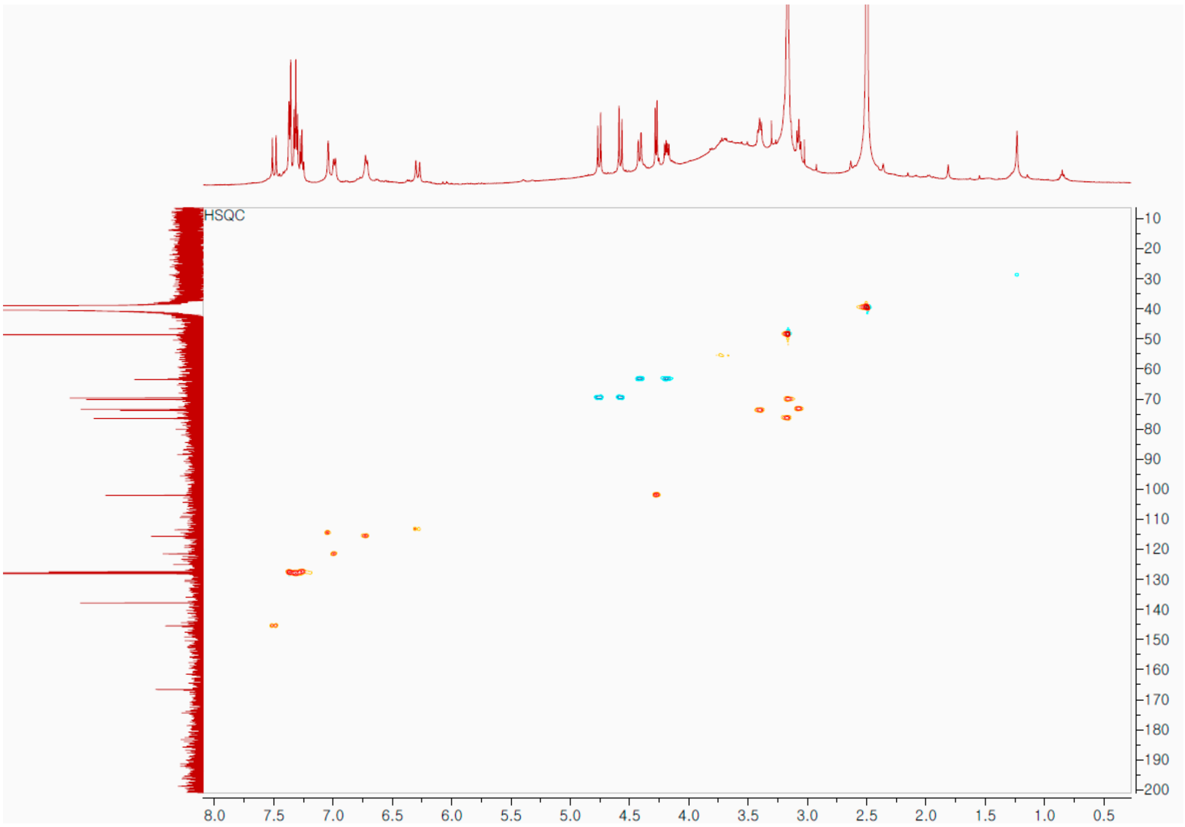

Figure S4. HSQC spectrum of compound **1** (CD_3OD, d ppm).

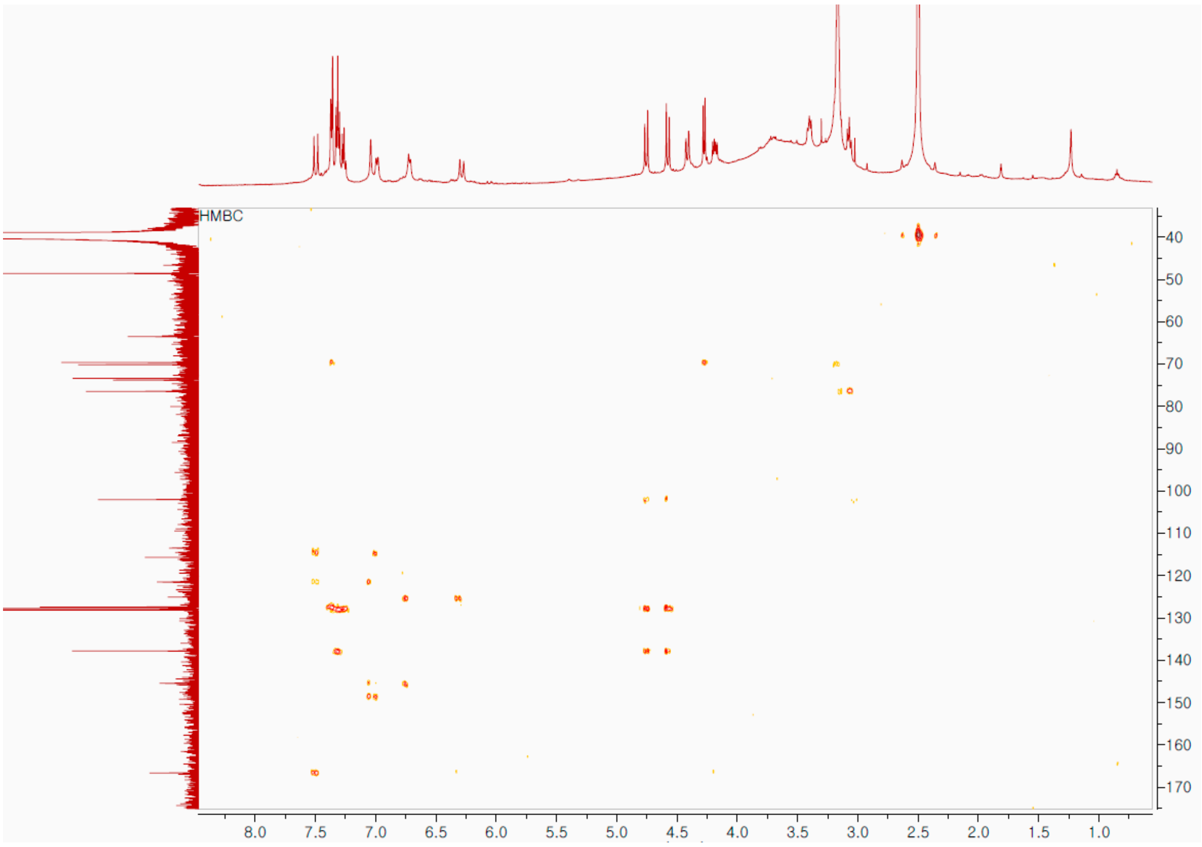

Figure S5. HMBC spectrum of compound **1** (CD_3OD, δ ppm).

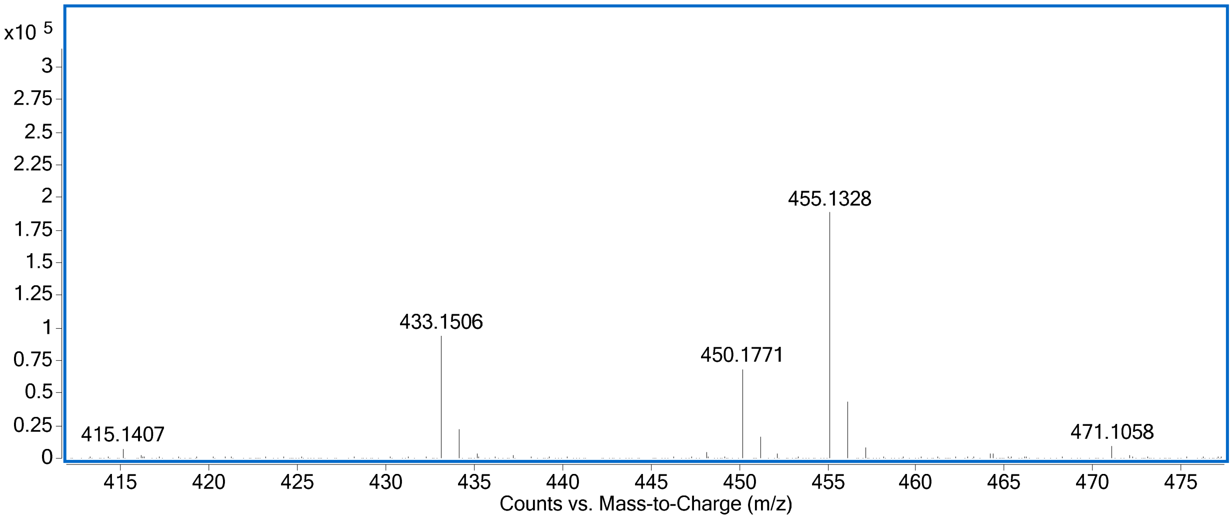

Figure S6. Q-TOF/MS spectrum of compound **1**.

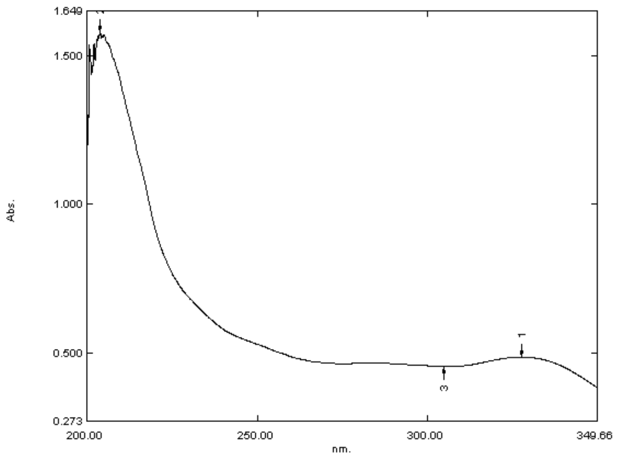

Figure S7. Ultraviolet spectrum of compound **1**.

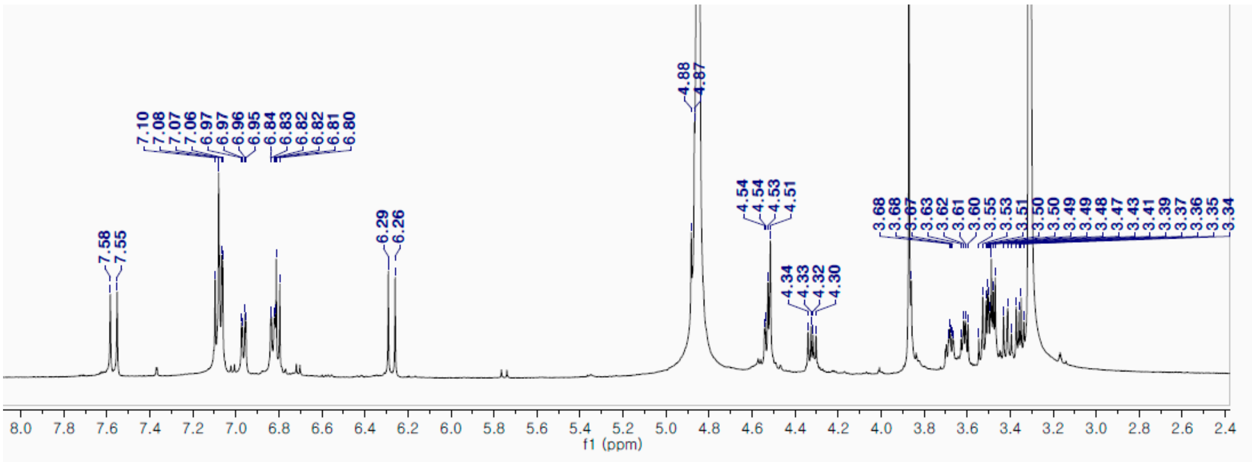

Figure S8. ^{1}H-NMR spectrum of compound **2** (CD_3OD, 500 MHz, δ ppm).

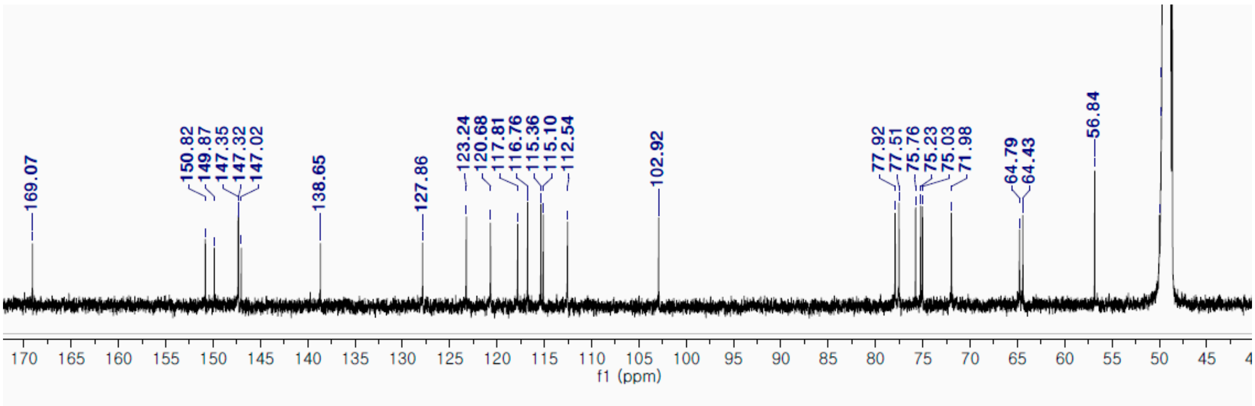

Figure S9. ^{13}C-NMR spectrum of compound **2** (CD_3OD, 125 MHz, δ ppm).

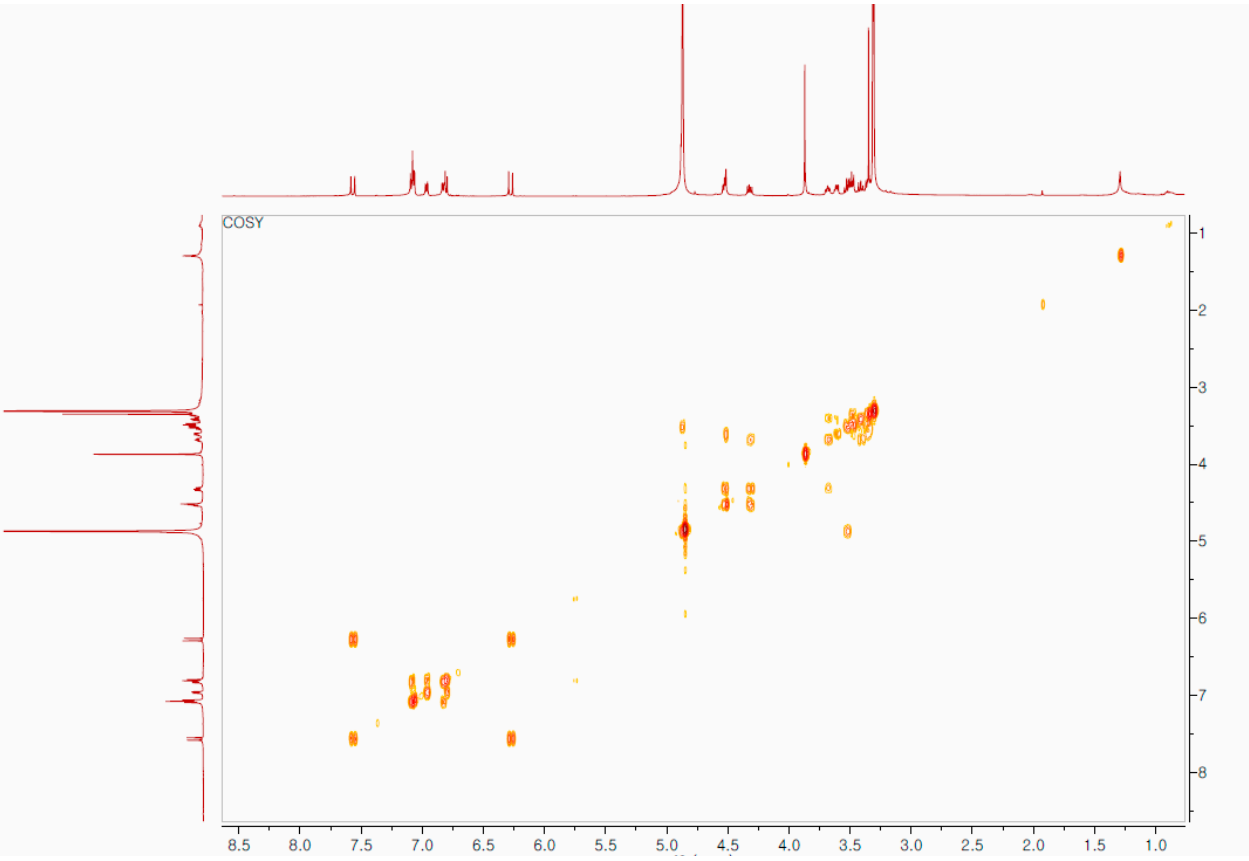

Figure S10. ^{1}H-^{1}H COSY spectrum of compound **2** (CD_3OD, δ ppm).

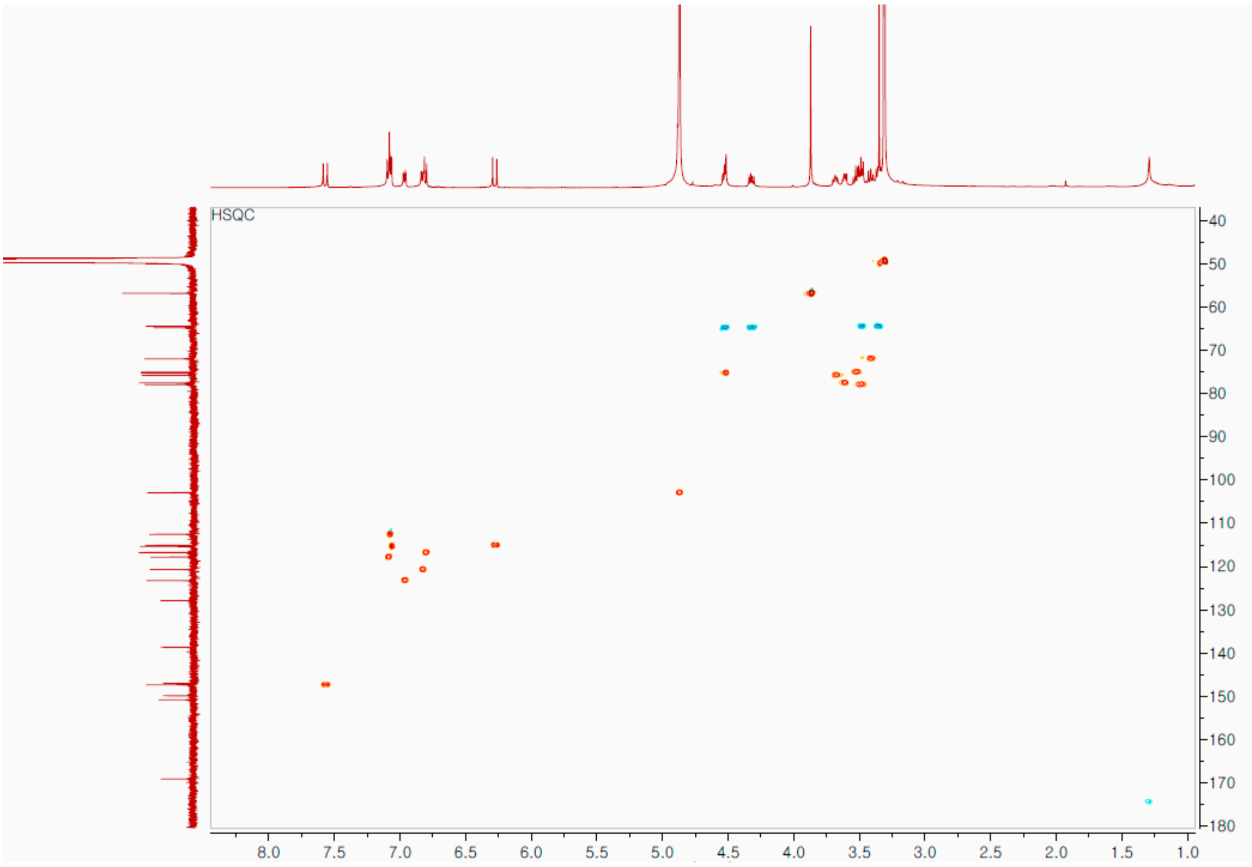

Figure S11. HSQC spectrum of compound **2** (CD_3OD, δ ppm).

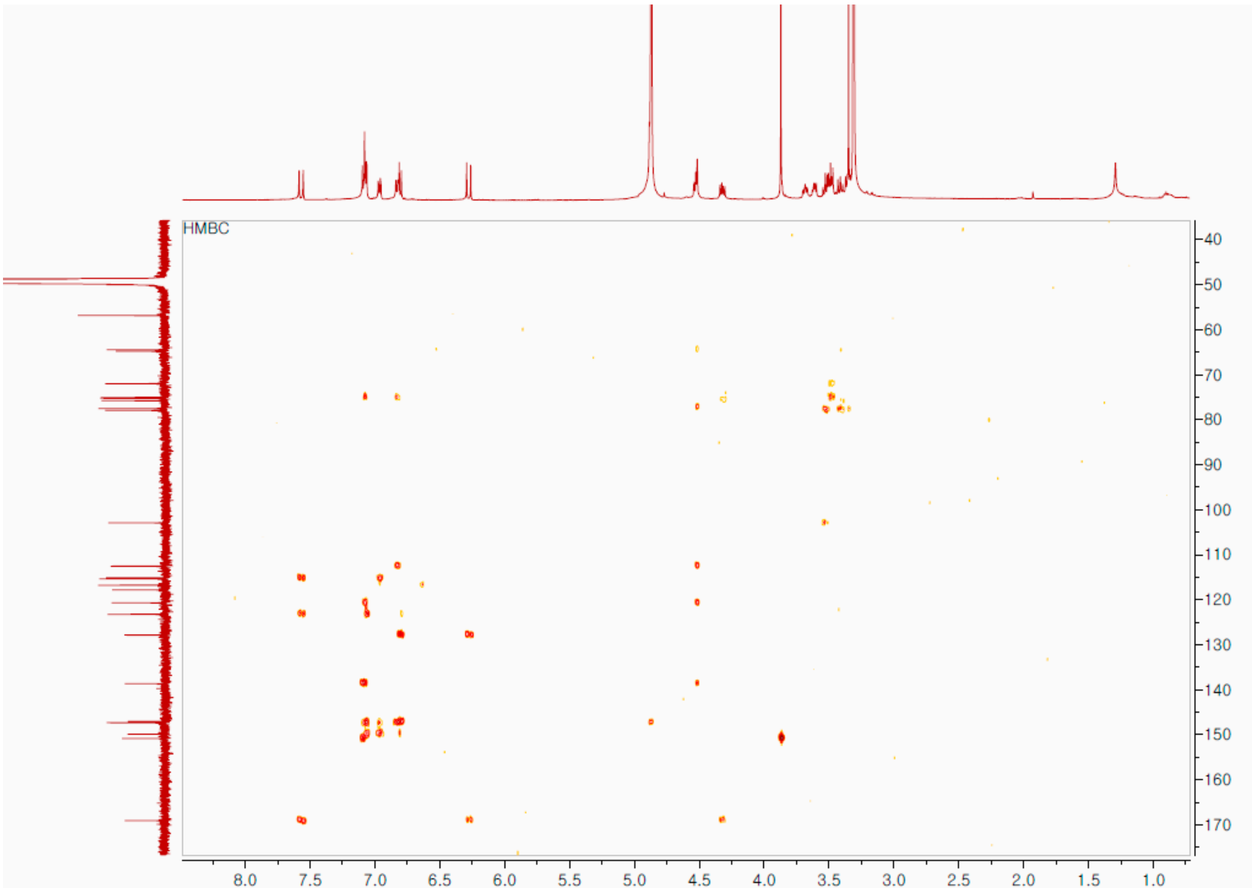

Figure S12. HMBC spectrum of compound **2** (CD_3OD, δ ppm).

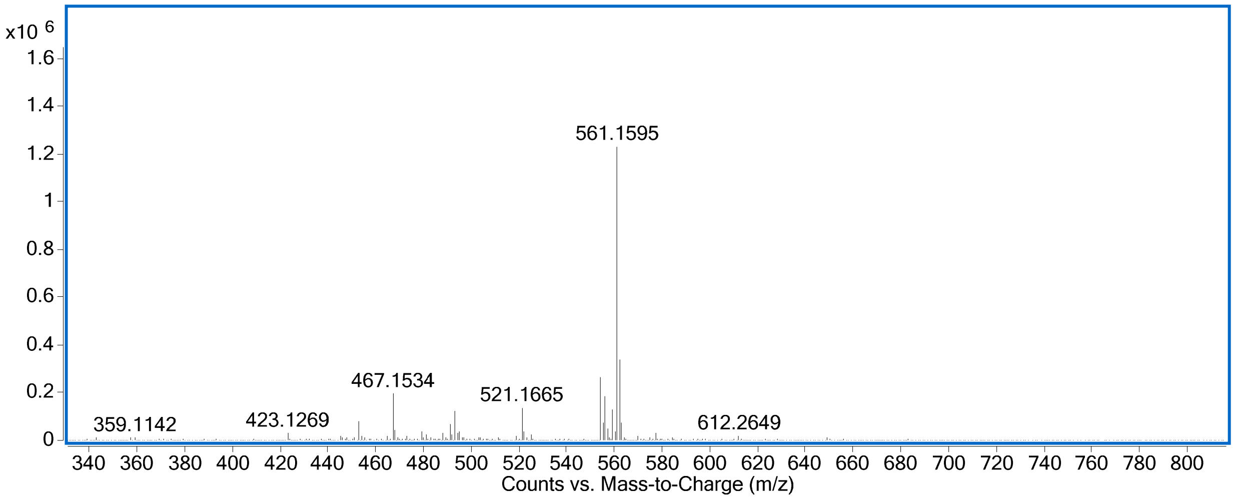

Figure S13. Q-TOF/MS spectrum of compound **2**.

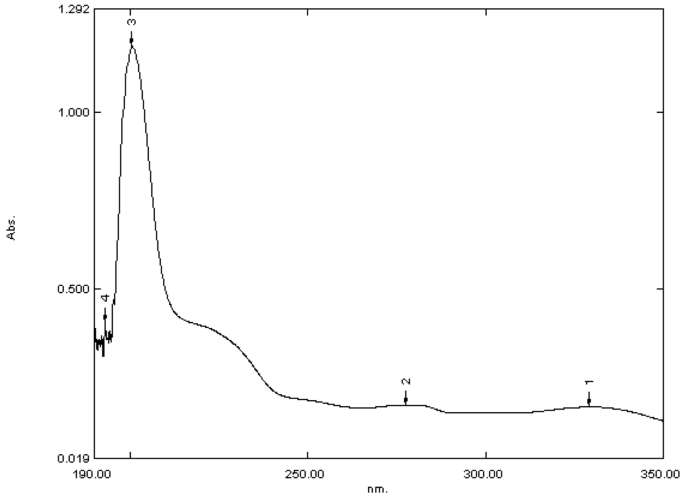

Figure S14. Ultraviolet spectrum of compound **2**.

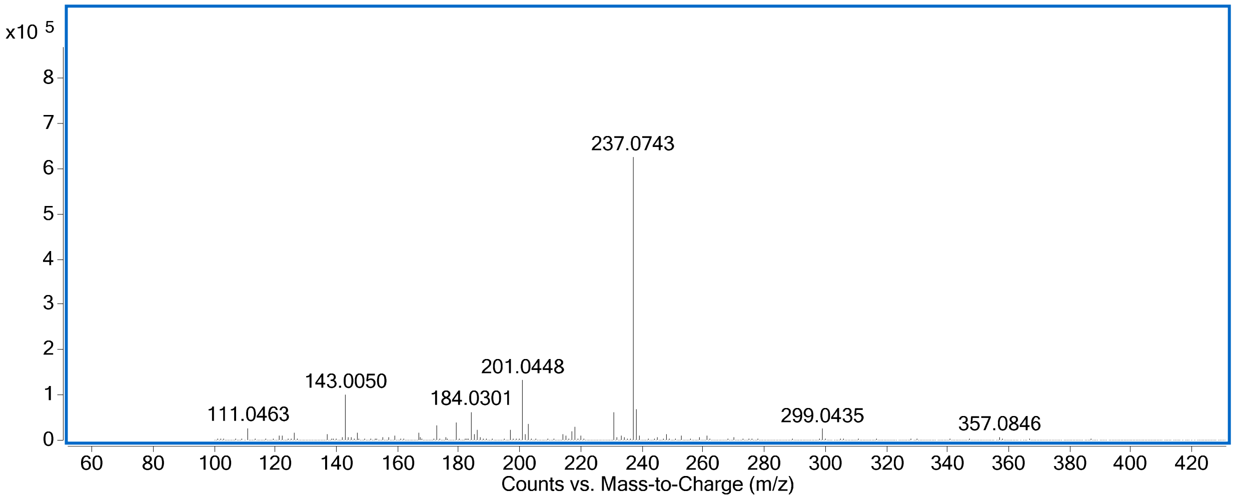

Figure S15. Q-TOF/MS spectrum of compound **2a**.

Spectroscopic Data of Compounds 1–12:

1-O-benzyl-6-O-E-caffeoyl-β-D-glucopyranoside (**1**): brown amorphous powder; Q-TOF/MS: *m/z* 433.1506 $[M+H]^+$ (calcd. for 433.1499, $C_{22}H_{25}O_9$), 455.1328 $[M+Na]^+$ (calcd. for 455.1318, $C_{22}H_{24}O_9Na$); $[\alpha]_D^{22}$ +21.36 (*c* 0.1, MeOH); UV$_{\lambda max}$ (MeOH) 203.9, 327.5 nm; ^{1}H- and ^{13}C-NMR, see Table 1 in the text.

1-O-(7S,8R)-guaiacylglycerol-(6-O-E-caffeoyl)-β-D-glucopyranoside (**2**): brown amorphous powder; Q-TOF/MS: *m/z* 561.1595 $[M+Na]^+$ ($C_{25}H_{30}O_{13}Na$, calcd for 561.1584); $[\alpha]_D^{22}$ −19.6 (*c* 0.1, MeOH); UV$_{\lambda max}$ (MeOH) 200.7, 224.4, 278.2, 330.8 nm; ^{1}H- and ^{13}C-NMR, see Table 1 in the text.

(7S,8R)-Guaiacylglycerol (**2a**): colorless amorphous powder; Q-TOF/MS: 237.0743 $[M+Na]^+$ ($C_{10}H_{14}O_5Na$, clacd for 237.0739); $[\alpha]_D^{22}$ +11.2 (*c* 0.02, MeOH).

Quercetin-3-O-β-D-galactopyranoside (**3**): yellow amorphous powder; Q-TOF/MS: *m/z* 465.1034 $[M+H]^+$ (calcd for $C_{21}H_{21}O_{12}$ 465.1033); ^{1}H-NMR (500 MHz, DMSO-*d*$_6$): δ 3.29 (1H, m, H-6"b), 3.31

(1H, m, H-3''), 3.35 (1H, m, H-2''), 3.46 (1H, m, H-6''a), 3.56 (1H, m, H-5''), 3.64 (1H, m, H-4''), 5.35 (1H, d, *J* = 7.6 Hz, H-1''), 6.14 (1H, s, H-6), 6.35 (1H, s, H-8), 6.80 (1H, d, *J* = 8.4 Hz, H-5'), 7.52 (1H, d, *J* = 1.7 Hz, H-2'), 7.66 (1H, dd, *J* = 8.4, 1.7 Hz, H-6'); ^{13}C-NMR (125 MHz, DMSO-d_6): δ 60.1 (C-6″), 67.9 (C-4''), 71.2 (C-2''), 73.2 (C-3''), 75.8 (C-5''), 93.6 (C-8), 99.0 (C-6), 101.9 (C-1''), 103.3 (C-10), 115.1 (C-5'), 115.8 (C-2'), 120.9 (C-6'), 121.9 (C-1'), 133.3 (C-3), 144.9 (C-3'), 148.6 (C-4'), 156.4 (C-9), 156.6 (C-2), 161.7 (C-5), 165.5 (C-7), 177.2 (C-4).

Quercetin-3-O-(6''-O-E-caffeoyl)-β-D-galactopyranoside (**4**): yellow amorphous powder; Q-TOF/MS: *m/z* 627.1349 [M+H]$^+$ (calcd for $C_{30}H_{27}O_{15}$ 627.1350); ^{1}H-NMR (500 MHz, DMSO-d_6): δ 3.42 (1H, m, H-3''), 3.58 (1H, m, H-2''), 3.66 (1H, m, H-4''), 3.67 (1H, m, H-5''), 4.02 (1H, m, H-6''b), 4.11 (1H, m, H-6''a), 5.40 (1H, d, *J* = 7.7 Hz, H-1″), 6.01 (1H, d, *J* = 15.9 Hz, H-8'''), 6.12 (1H, s, H-6), 6.33 (1H, s, H-8), 6.73 (1H, d, *J* = 8.1 Hz, H-5'''), 6.80 (1H, dd, *J* = 7.7, 1.7 Hz, H-6'''), 6.82 (1H, d, *J* = 8.4 Hz, H-5'), 6.93 (1H, d, *J* = 1.7 Hz, H-2'''), 7.30 (1H, d, *J* = 15.9 Hz, H-7'''), 7.51 (1H, d, *J* = 2.1 Hz, H-2'), 7.64 (1H, dd, *J* = 8.4, 2.1 Hz, H-6'); ^{13}C-NMR (125 MHz, DMSO-d_6): δ 63.5 (C-6''), 68.7 (C-4''), 71.5 (C-2''), 73.3 (C-3''), 73.3 (C-5''), 94.0 (C-8), 99.4 (C-6), 102.0 (C-1''), 103.7 (C-10), 113.6 (C-8'''), 115.0 (C-2'''), 115.7 (C-2'), 116.0 (C-5'''), 116.3 (C-5'), 121.5 (C-6'), 121.7 (C-6'''), 122.3 (C-1'), 125.6 (C-1'''), 133.8 (C-3), 145.2 (C-3'), 145.2 (C-7'''), 146.1 (C-3'''), 148.8 (C-4'), 148.9 (C-4'''), 156.7 (C-2), 156.7 (C-9), 161.5 (C-5), 166.5 (C-9'''), 166.6 (C-7), 177.7 (C-4).

1,6-Dicaffeoyl-β-D-glucopyranoside (**5**): yellow amorphous powder; Q-TOF/MS: *m/z* 527.1176 [M+Na]$^+$ (calcd for $C_{24}H_{24}O_{13}Na$ 527.1165); ^{1}H-NMR (500 MHz, CD_3OD): δ 3.42 (1H, m, H-4''), 3.44 (1H, m, H-2'), 3.47 (1H, m, H-5'), 3.66 (1H, m, H-3'), 4.31 (1H, m, H-6''b), 4.50 (1H, m, H-6''a), 5.59 (1H, d, *J* = 7.7 Hz, H-1'), 6.29 (1H, d, *J* = 15.8 Hz, H-7''), 6.31 (1H, d, *J* = 15.9 Hz, H-7), 6.77 (1H, d, *J* = 8.2 Hz, H-5''), 6.78 (1H, d, *J* = 8.2 Hz, H-5), 6.95 (1H, dd, *J* = 6.1, 1.9 Hz, H-6''), 6.97 (1H, dd, *J* = 6.1, 1.9 Hz, H-6), 7.05 (1H, d, *J* = 1.9 Hz, H-2''), 7.06 (1H, d, *J* = 1.9 Hz, H-2), 7.57 (1H, d, *J* = 15.9 Hz, H-8''), 7.66 (1H, d, *J* = 15.9 Hz, H-8); ^{13}C-NMR (125 MHz, CD_3OD): δ 64.2 (C-6'), 71.3 (C-4'), 73.9 (C-2'), 76.3 (C-3'), 77.8 (C-5'), 95.7 (C-1'), 114.2 (C-7), 114.7 (C-7''), 115.2 (C-2), 115.2 (C-2''), 116.4 (C-5), 116.4 (C-5''), 122.9 (C-6), 123.0 (C-6''), 127.6 (C-1), 127.9 (C-1''), 147.0 (C-3''), 147.2 (C-3), 147.3 (C-8''), 148.4 (C-8), 149.6 (C-4''), 149.7 (C-4), 167.4 (C-9), 169.1 (C-9'').

Benzyl 6'-O-galloyl-β-D-glucopyranoside (**6**): brown amorphous powder; Q-TOF/MS: *m/z* 445.1109 [M+Na]$^+$ (calcd for $C_{20}H_{22}O_{10}Na$ 445.1111); ^{1}H-NMR (500 MHz, DMSO-d_6): δ 3.10 (1H, m, H-2'), 3.20 (1H, m, H-3'), 3.24 (1H, m, H-4'), 3.42 (1H, m, H-5'), 4.28 (1H, m, H-6'b), 4.29 (1H, d, *J* = 7.8 Hz, H-1'), 4.46 (1H, m, H-6'a), 4.57 (1H, d, *J* = 12.0 Hz, H-7b), 4.75 (1H, d, *J* = 12.0 Hz, H-7a), 7.00 (2H, s, H-2'', 6''), 7.28 (1H, m, H-4), 7.32 (2H, m, H-3, 5), 7.36 (2H, m, H-2, 6); ^{13}C-NMR (125 MHz, DMSO-d_6): δ 63.0 (C-6'), 69.5 (C-7), 70.0 (C-4'), 73.1 (C-2'), 73.8 (C-5'), 76.4 (C-3'), 102.0 (C-1'), 108.5 (C-2''), 108.5 (C-6''), 120.5 (C-1''), 127.3 (C-4), 127.7 (C-2), 127.7 (C-6), 128.1 (C-3), 128.1 (C-5), 137.6 (C-1), 138.4 (C-4''), 145.4 (C-3''), 145.4 (C-5''), 165.7 (C-7'').

Diohydrosyringin (**7**): white amorphous powder; Q-TOF/MS: *m/z* 397.1480 [M+Na]$^+$ (calcd for $C_{17}H_{26}O_9Na$ 397.1475); ^{1}H-NMR (500 MHz, CD_3OD): δ 1.83 (2H, m, H-8), 2.64 (2H, t, *J* = 7.4 Hz, H-7), 3.20-3.46 (4H, m, H-2'-6'), 3.57 (1H, t, *J* = 6.4 Hz, H-9), 3.67 (1H, m, H-6'b), 3.79 (1H, m, H-6'a), 3.82 (3H, s, -OMe), 4.80 (1H, d, *J* = 7.5 Hz, H-1'), 6.56 (2H, s, H-2, 6); ^{13}C-NMR (125 MHz, CD_3OD): δ

33.5 (C-7), 35.5 (C-8), 62.2 (-OCH3), 62.3 (C-9), 62.8 (C-6'), 71.4 (C-4'), 75.9 (C-2'), 77.9 (C-3'), 78.4 (C-5'), 105.8 (C-1'), 107.6 (C-2), 107.6 (C-6), 135.4 (C-4), 140.6 (C-1), 154.2 (C-3), 154.2 (C-5).

1-O-E-caffeoyl-β-D-glucopyranoside (**8**): brown amorphous powder; Q-TOF/MS: *m/z* 365.0847 $[M+Na]^+$ (calcd for $C_{15}H_{18}O_9Na$ 365.0849); ^{1}H-NMR (500 MHz, CD_3OD): δ 3.38 (1H, m, H-4'), 3.40 (1H, m, H-5'), 3.42 (1H, m, H-2'), 3.45 (1H, m, H-3'), 3.69 (1H, m, H-6'b), 3.85 (1H, m, H-6'a), 5.57 (1H, d, *J* = 7.8 Hz, H-1'), 6.30 (1H, d, *J* = 15.7 Hz, H-7), 6.79 (1H, d, *J* = 8.1 Hz, H-5), 6.97 (1H, dd, *J* = 8.1, 1.7 Hz, H-6), 7.06 (1H, d, *J* = 1.7 Hz, H-4), 7.66 (1H, d, *J* = 15.7 Hz, H-8); ^{13}C-NMR (125 MHz, CD_3OD): δ 60.9 (C-6'), 69.7 (C-4'), 72.6 (C-2'), 76.6 (C-3'), 77.4 (C-5'), 94.3 (C-1'), 112.9 (C-7), 113.8 (C-2), 115.1 (C-5), 121.8 (C-1), 121.8 (C-6), 145.4 (C-3), 146.9 (C-8), 148.7 (C-4), 166.4 (C-9).

3-O-Methylellagic aicd 4'-sulfate (**9**): yellow amorphous powder; Q-TOF/MS: *m/z* 396.9876 $[M+H]^+$ (calcd for $C_{15}H_9O_{11}S$ 396.9866); ^{1}H-NMR (500 MHz, DMSO-d_6): δ 3.99 (3H, s, -OMe), 7.34 (1H, s, H-5), 7.64 (1H, s, H-5'); ^{13}C-NMR (125 MHz, DMSO-d_6): δ 60.0 (-OMe), 107.4 (C-6'), 111.1 (C-1), 111.1 (C-5), 113.9 (C-6), 116.4 (C-1'), 118.7 (C-5'), 137.5 (C-2'), 140.6 (C-3), 142.5 (C-2), 142.5 (C-4'), 145.5 (C-3'), 152.0 (C-4), 159.8 (C-7'), 160.5 (C-7).

3-O-Methylellagic acid (**10**): yellow amorphous powder; Q-TOF/MS: *m/z* 317.0294 $[M+H]^+$ (calcd for $C_{15}H_9O_8$ 317.0297); ^{1}H-NMR (500 MHz, DMSO-d_6): δ 4.03 (3H, s, -OMe), 7.44 (1H, s, H-5'), 7.52 (1H, s, H-5); ^{13}C-NMR (125 MHz, DMSO-d_6): δ 60.8 (-OMe), 106.9 (C-6'), 110.2 (C-5'), 111.2 (C-5), 111.8 (C-1), 112.4 (C-6), 113.2 (C-1'), 136.1 (C-2'), 140.0 (C-3'), 140.1 (C-3), 141.5 (C-2), 148.3 (C-4'), 152.7 (C-4), 158.8 (C-7'), 158.9 (C-7).

Chlorogenic acid (**11**): brown amorphous powder; Q-TOF/MS: *m/z* 355.1039 $[M+H]^+$ (calcd for $C_{16}H_{19}O_9$ 355.1029); ^{1}H-NMR (500 MHz, DMSO-d_6): δ 1.88-1.47 (4H, m, H-2, 6), 3.58 (1H, m, H-3), 3.64 (2H, m, H-4, 5), 6.20 (1H, d, *J* = 15.6 Hz, H-8'), 6.74 (1H, d, *J* = 7.5 Hz, H-5'), 6.98 (1H, d, *J* = 7.5 Hz, H-6'), 7.03 (1H, d, *J* = 1.5 Hz, H-2'), 7.48 (1H, d, *J* = 15.6 Hz, H-7'); ^{13}C-NMR (125 MHz, DMSO-d_6): δ 37.0 (C-2), 39.9 (C-6), 69.8 (C-3), 70.6 (C-5), 70.8 (C-4), 73.8 (C-1), 114.9 (C-2'), 115.0 (C-8'), 116.2 (C-5'), 121.6 (C-6'), 125.7 (C-1'), 145.1 (C-7'), 146.2 (C-3'), 149.6 (C-4'), 166.8 (C-9'), 179.4 (C-7).

Cryptochlorogenic acid (**12**): brown amorphous powder; Q-TOF/MS: *m/z* 355.1019 $[M+H]^+$ (calcd for $C_{16}H_{19}O_9$ 355.1029); ^{1}H-NMR (500 MHz, DMSO-d_6): δ 1.84-2.21 (4H, m, H-2, 6), 4.13 (2H, m, H-3, 5), 4.79 (1H, d, *J* = 2.9 Hz, H-4), 6.24 (1H, d, *J* = 15.8 Hz, H-8'), 6.74 (1H, d, *J* = 8.1 Hz, H-5'), 6.98 (1H, dd, *J* = 8.1, 1.5 Hz, H-6'), 7.04 (1H, d, *J* = 1.5 Hz, H-2'), 7.45 (1H, d, *J* = 15.8 Hz, H-7'); ^{13}C-NMR (125 MHz, DMSO-d_6): δ 37.0 (C-2), 41.5 (C-6), 64.0 (C-5), 70.0 (C-3), 75.4 (C-1), 79.0 (C-4), 114.5 (C-2'), 114.6 (C-8'), 115.7 (C-5'), 121.1 (C-6'), 125.7 (C-1'), 144.6 (C-3'), 145.6 (C-7'), 148.6 (C-4'), 166.2 (C-9'), 178.7 (C-7).
